# The Process and Mechanism of Preparing Nanoporous Silicon: Helium Ion Implantation

**DOI:** 10.3390/nano13081324

**Published:** 2023-04-10

**Authors:** Jianguang Wang, Kelin Zhu, Xiaoling Wu, Guoan Cheng, Ruiting Zheng

**Affiliations:** Key Laboratory of Radiation Beam Technology and Materials Modification of the Ministry of Education, College of Nuclear Science and Technology, Beijing Normal University, Beijing 100875, China; jgwang@mail.bnu.edu.cn (J.W.);

**Keywords:** porous silicon, 1~55 nm diameter helium bubble, helium ion implantation, mechanism of growth

## Abstract

Ion implantation is an effective way to control performance in semiconductor technology. In this paper, the fabrication of 1~5 nm porous silicon by helium ion implantation was systemically studied, and the growth mechanism and regulation mechanism of helium bubbles in monocrystalline silicon at low temperatures were revealed. In this work, 100 keV He ions (1~7.5 × 10^16^ ions/cm^2^) were implanted into monocrystalline silicon at 115 °C~220 °C. There were three distinct stages in the growth of helium bubbles, showing different mechanisms of helium bubble formation. The minimum average diameter of a helium bubble is approximately 2.3 nm, and the maximum number density of the helium bubble is 4.2 × 10^23^ m^−3^ at 175 °C. The porous structure may not be obtained at injection temperatures below 115 °C or injection doses below 2.5 × 10^16^ ions/cm^2^. In the process, both the ion implantation temperature and ion implantation dose affect the growth of helium bubbles in monocrystalline silicon. Our findings suggest an effective approach to the fabrication of 1~5 nm nanoporous silicon, challenging the classic view of the relationship between process temperature or dose and pore size of porous silicon, and some new theories are summarized.

## 1. Introduction

Porous silicon (PS) is a new type of functional material with a sponge structure based on nano-silicon grains. With its superior properties, PS has shown good applications in many fields. For example, porous silicon is used in optoelectronic devices because of its photoluminescence properties [1,2]. At the same time, porous silicon is a kind of natural sensor material because of its great inner surface area and unique electrical properties [3,4,5,6]. In addition, the cavity structure of porous silicon can scatter the heat conduction phonons, which is expected to be used to develop new thermoelectric materials [7,8,9].

Therefore, the preparation of porous silicon has always been the focus of attention from scientific researchers. The traditional method is to form porous silicon on the silicon surface by chemical or electrochemical etching [10,11,12,13]. A novel preparation method was reported by the Yang group in 2010, they use nanosphere or block-copolymer lithography with deep reactive ion etching on thin monocrystalline silicon films to prepare porous silicon with uniform pore spacing of 55,140 or 350 nm [9,14]. Liu et al. reported the method of Electron Beam Lithography (EBL) and deep reactive ion etching (DRIE) single crystal Si nano barrel with diameters ranging from 100 to 300 nm [15].

All of which aim to control various parameters of the Si structures. Ion implantation has the advantages of precise control of experimental parameters and simple operation. Although the current reports on helium ion implantation in silicon, it is mainly used for proximity gettering of transition metal impurities [16], reduction of dislocation density [17], etc., we think it is a potential method in the preparation of porous silicon. Helium ion implantation in silicon can produce a nanocavity structure. For example, Raineria et al. investigate cavities and bubbles formed in silicon after high-dose helium implantation and high-temperature annealing in 1995 [16]; the basic mechanisms responsible for the formation and growth of such structures in single-crystalline silicon were reported by Cerofolini et al.in 2000 [18]. M. L. David et al. investigate the effect of implant temperature on defects created using a high fluence of helium in silicon in 2003 [19]. The formation and growth of defects, including nanocavities and extended interstitial-type defects, created by helium implantation in silicon were continuously investigated by D. Babonneau et al. using grazing incidence small-angle x-ray scattering technique in 2006 [20], the L. Pizzagalli group using Electron Energy-loss Spectroscopy technique in 2011 [21], and Ono et al. using situ TEM, STEM-EELS, and TDS technique in 2019 [22]. 

However, most of the pore size distributions are in the range of 10 nm~100 µm in most of the reports on porous silicon, and very little attention has been given to pore sizes below 5 nm. According to our investigation, porous silicon with small pore size has good application. According to Lee et al., well-ordered nanoporous silicon materials with pore sizes between 0.6 nm and 1.2 nm and porosity between 12% and 30% exhibit higher thermoelectric properties [7,8]. In particular, although there are many reports on the growth mechanism of helium bubbles at high temperatures, the growth mechanism at low temperatures is still not clear enough. However, low-temperature ion implantation is the key to the preparation of porous silicon with a small pore size.

Under this condition, we mainly focus on the preparation of porous silicon with a pore size below 5 nm by ion implantation. the growth rule and mechanism of helium bubble pore structure in monocrystalline silicon at low temperatures are also revealed. The research will help us to understand the process of ion beams regulating the microstructure of silicon-based materials, help to develop new functional materials and expand the application fields of ion implantation technology.

## 2. Materials and Methods

The original sample was a 100 mm diameter p-type B-doped Czochralski (100) silicon wafer. The resistivity of the sample was 1~5 m Ω m, and the thickness was 500 ± 15 µm. After being cleaned by the standard RCA process, helium ions were injected into the sample. The injection criteria are shown in Table 1.

Transmission electron microscopy (Talos f200x G2 TEM) was used to observe the cross-section of the silicon wafer. The cross-section TEM slices were prepared by focused ion beam (FIB) stripping technology, and the slice thickness was approximately 50 nm. To highlight the contrast between the cavity edge and FRESNEL, we need imaging under the condition of underfocus. We obtain the “average helium bubble diameter” and “helium bubble number density distribution” by the following method: (1). The diameter of the helium bubble and defect layer was measured using the Nano Measurer software. (2). We count the number of helium bubbles in a certain area of the electron microscope image, and the thickness of the electron microscope sample is known to be 40 nm. Then, the density of the helium bubble can be obtained through the formula calculation. Its accuracy mainly depends on the resolution of the electron microscope and the accuracy of the thickness of the sample. (3). From multiple samples, the average is obtained.

## 3. Results

**Figure 1:**
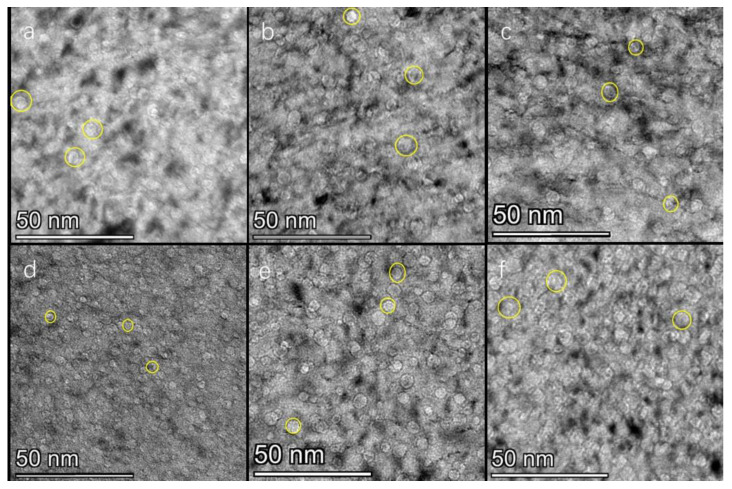
Cross-sectional TEM micrograph of He ion-implanted into single crystal Si (100 keV, dose 5 × 10^16^ ions/cm^2^) at different sample temperatures (**a**) 115 °C, (**b**) 145 °C, (**c**) 160 °C, (**d**) 175 °C, (**e**) 190 °C, and (**f**) 220 °C.

Figure 1 shows TEM images of p-type monocrystalline silicon (5 × 10^16^ ions/cm^2^ dose) implanted with 100 Kev helium ions at 115 °C~220 °C. As shown in Figure 1a–f, it is clear that the helium bubbles are uniform in shape, resembling a round bubble, and a defect layer is formed below the surface of the silicon wafer when a large number of helium bubbles accumulated. The average diameter of the helium bubble is approximately 3 nm, and the width of the defect layer is approximately 450 nm. Specific parameters are shown in Table 1. A large number of flaky defects can be seen in the defect layer, and such defects are considered to be interstitial defects that have been described in many works in the literature, sheet-like or rod-like defects [17,23,24,25,26], which are silicon-helium complexes formed by the precipitation of excess helium atoms in the tetrahedral interstitial position of the silicon lattice. As seen from the section, the length, and thickness of this defect are approximately 15–60 nm and 1 nm, respectively. The electron diffraction pattern shows that the crystal lattice is not destroyed periodically, most of them are parallel to the surface of silicon, and the spacing is approximately 6 nm. As shown in Figure 1b, there are a large number of flaky defects in the defect layer. Figure 1d shows that the average diameter of the helium bubble is approximately 2.3 nm. Compared with the two figures above, the diameter of the helium bubble grown by this process is smaller, and no sheet-like or rod-like defects are observed in the defect layer. Compared with Figure 1d, the diameter of the helium bubble grown in this process is doubled in Figure 1f.

**Figure 2:**
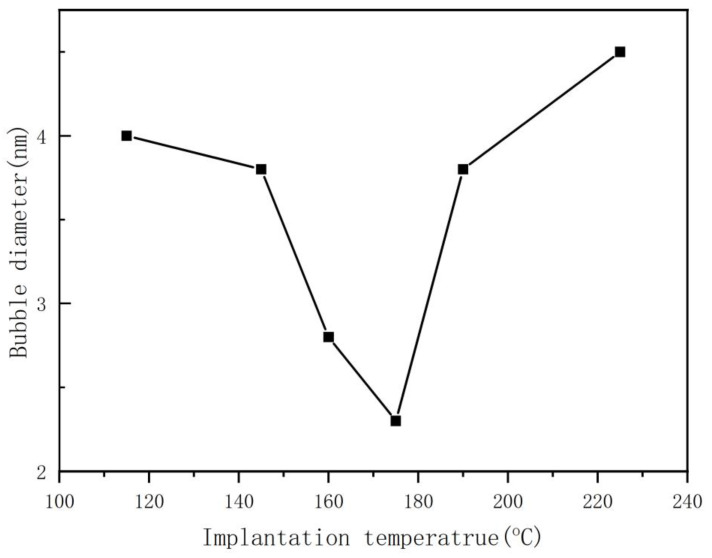
Dependence of the average helium bubble diameter on the helium ion implantation temperature (helium ion dose 5 × 10^16^ ions/cm_2_, helium ion energy 100 KeV).

**Figure 3:**
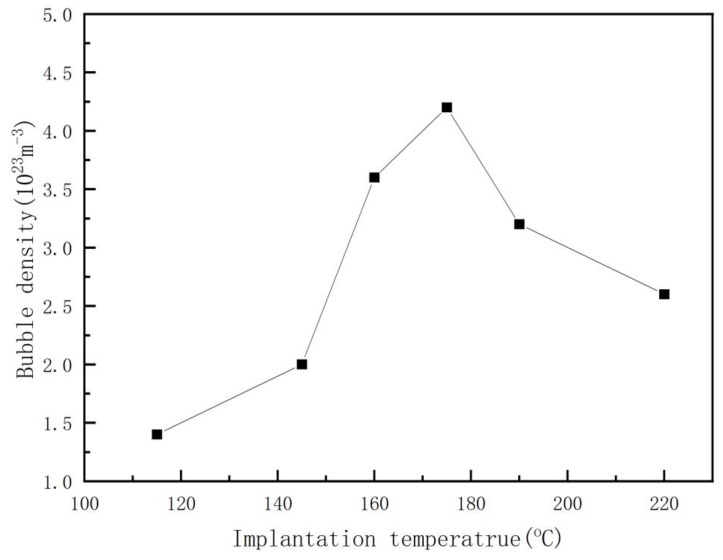
Dependence of the helium bubble number density distribution on the helium ion implantation temperature (helium ion dose 5 × 10^16^ ions/cm^2^, helium ion energy 100 KeV).

The relationship between the average diameter of the helium bubble and the helium ion implantation temperature is not simply a positive or negative correlation, as is clear from Figure 2, a phenomenon that has not been mentioned in other reports. According to the existing theory, the mobility of helium ions and vacancies in the silicon matrix increases with increasing process temperature, which is more favorable to the growth of the helium bubble, and the diameter of the helium bubble increases with increasing process temperature. However, it is found that the diameter of the helium bubble decreases with an increase in the process temperature below 175 °C. This should be related to the appearance of sheet-like defects. In our experiment, a large number of sheet-like defects were observed by electron microscopy at 115 °C and 145 °C. The occurrence of sheet-like defects affects the nucleation of the helium bubble for reasons we will analyze in the following sections. At the same dose of helium ion implantation, the density of helium bubbles changes with increasing implantation temperature, which is closely related to the variation in the diameter of the helium bubble, which can be seen clearly in Figure 2 and Figure 3. The calculated helium bubble number density is lower than the actual density because very small helium bubbles may not be observed under electron microscopy.

**Figure 4:**
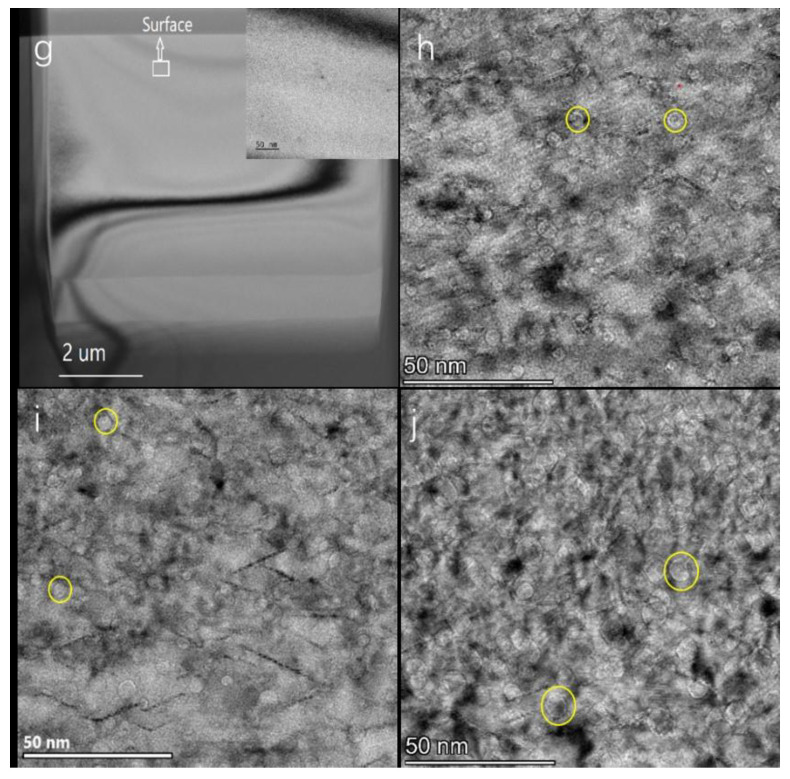
TEM images of different helium ion (100 Kev) doses at 180°, (**g**) 1 × 10^16^ ions/cm^2^, the enlarged area (inset) indicates that no helium bubble structure was observed, (**h**) 2.5 × 10^16^ ions/cm^2^, (**i**) 5 × 10^16^ ions/cm^2^, and (**j**) 7.5 × 10^16^ ions/cm^2^ implanted into monocrystalline silicon.

In addition, it is found that the diameter of the helium bubble decreases at the edge of the defect layer, which suggests that the helium atom concentration is closely related to the size of the bubble. To test this idea, we designed a control group of helium ion implantation doses with dose distributions ranging from 1 × 10^16^ ions/cm^2^ to 7.5 × 10^16^ ions/cm^2^. Figure 4 shows TEM images of monocrystalline silicon implanted with 100 KeV helium ions (dose 1~7.5 × 10^16^ ions/cm^2^) at 180 °C. No helium bubbles were observed in Figure 4g, indicating that there is a critical concentration for the formation of helium bubbles in monocrystalline silicon, which is between 1 × 10^16^ ions/cm^2^ and 2.5 × 10^16^ ions/cm^2^, rather than the critical dose of 1 × 10^16^ ions/cm^2^ reported in the literature for helium bubble formation [16]. As seen clearly in Figure 4h–j the helium bubble is also uniformly circular, with a large number of helium bubbles distributed in a defect layer approximately 450 nm wide. The average diameter of the helium bubble is approximately 3.0 nm, and the specific parameters are shown in Table 1.

**Figure 5:**
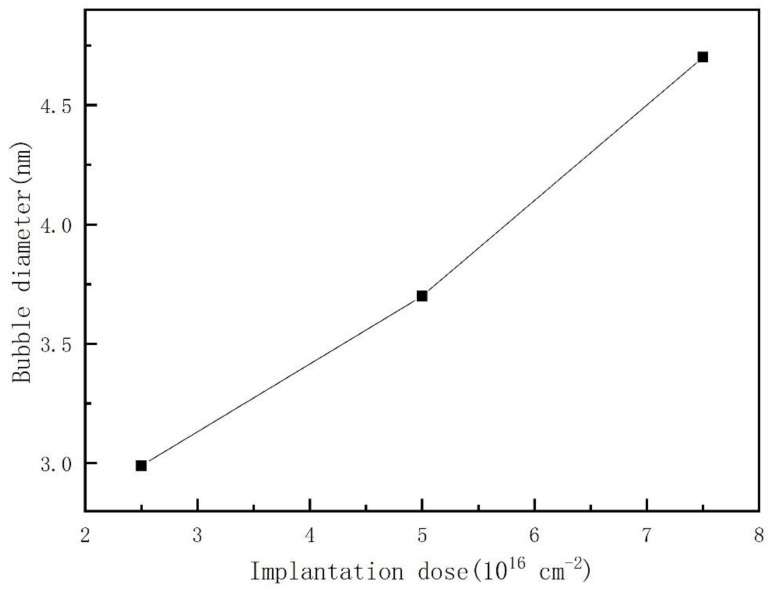
The relationship between the average diameter of helium bubbles and the helium ion implantation dose (helium ion implantation temperature of 180 °C and helium ion of 100 keV).

**Figure 6:**
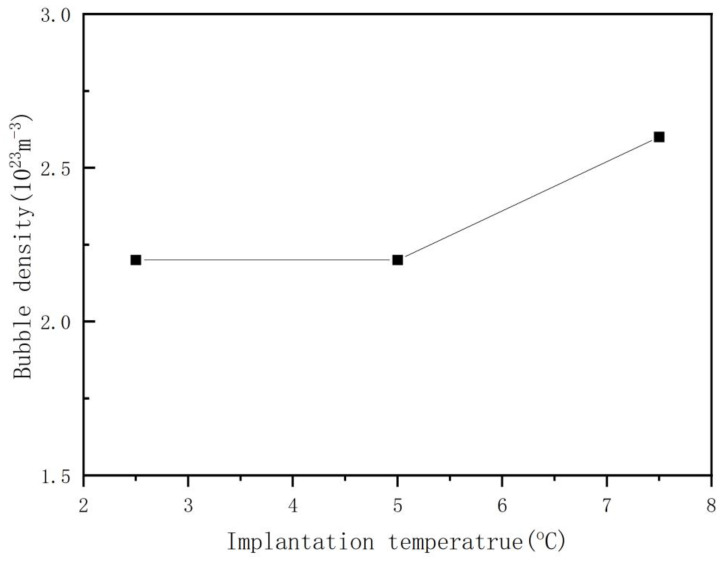
The relationship between helium bubble number density and helium ion implantation dose (helium ion implantation temperature of 180 °C, helium ion of 100 keV).

The relationship between the average diameter of the helium bubble and the helium ion implantation dose is shown in Figure 5. The diameter of the helium bubble increases with increasing helium ion implantation dose. This phenomenon is not common in similar reports, for example, Raineri and M. Luysberg think that the diameter of a helium bubble has little relationship with the helium ion implantation dose, and the helium ion implantation dose mainly affects the number density of the helium bubble [16,27]. However, from the data of Figure 5 and Figure 6, with increasing helium ion implantation dose, both the number density and the diameter of helium bubbles increase in our experiment. Compared with 2.5 × 10^16^ ions/cm^2^, the diameter of samples with a dose of 7.5 × 10^16^ ions/cm^2^ increased by 57% and 33%, respectively. This finding suggests that the growth of helium bubbles is closely related to the concentration of surrounding helium ions in the selected ion implantation temperature range (115 °C~220 °C).

The above experimental results can be visually shown in Table 1, which describes how different experimental processes specifically regulate the growth of helium bubbles.

## 4. Discussion

The growth process of a helium bubble is similar to that of a crystal, which generally goes through three stages: supersaturation, nucleation, and growth. According to the correlation between helium diffusion and temperature in some reports, the helium diffusion mechanism is divided into nonthermal mechanisms at low temperatures, including (1) the self-trapping/self-induction mechanism, (2) the mechanism of punching out dislocation loops, and (3) interstitial displacement mechanism, and the mechanism of thermal dissociation at high temperature. In the nonthermal mechanism, the nucleation of helium bubbles is diatomic nucleation [28], which generally occurs at low temperatures and high helium concentrations [29]. When the vacancy concentration is much smaller than the helium concentration, helium atoms may pass through the interstitial sites. However, the highly dispersed vacancies will strongly hinder helium diffusion through the interstitial mechanism, and the combination of helium atoms and vacancies is the most favorable way to reduce the energy of the system, so the vacancies can strongly trap helium atoms and form helium-vacancy complexes and then grow into helium bubbles. Because of the overpressure state of the small-sized helium bubbles, the pressure of the surrounding lattice distortion causes the helium bubbles to undergo heat-free migration and coalescence by “pushing out of the dislocation loop”. The interstitial displacement mechanism is controlled by the diffusion of helium through the self-interstitial/He displacement mechanism [30]. This mechanism is a recombination mechanism, which can be seen as an extension of the interstitial migration mechanism of helium atoms. Due to self-interstitial/vacancy recombination, the helium atoms trapped at the vacancies are emitted to the interstitial sites and migrate through the interstitial sites. The occurrence of this mechanism requires a certain activation energy, which is close to the vacancy migration energy at lower temperatures, similar to the case of point defect recombination [29]. The thermal dissociation mechanism occurs at higher temperatures (T > T_m_/2) [30,31], and the nucleation mechanism of helium bubbles is polyatomic nucleation. Only bubble nuclei exceeding the critical value can finally form helium bubbles, which are controlled by the thermal dissociation of helium atoms from the helium bubble nucleus [30]. The activation energy is related to the helium dissociation energy [32,33], and the thermal dissociation mechanism of the helium bubble growth rate is greater than the self-interstitial displacement rate.

**Figure 7:**
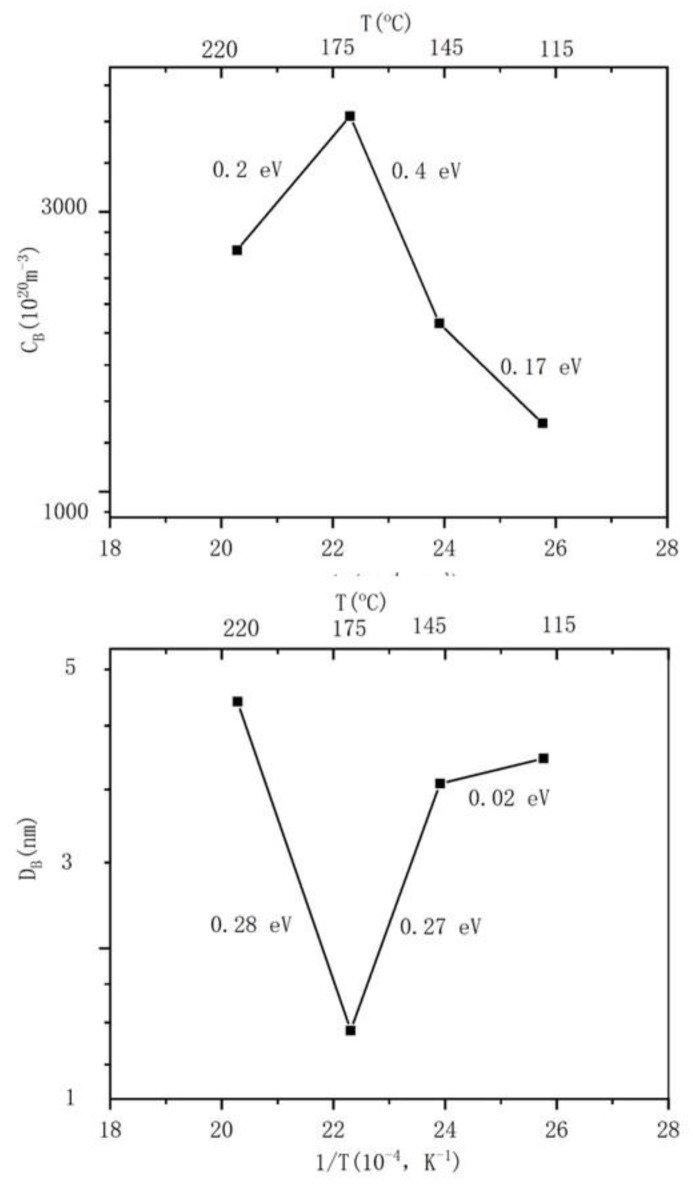
Temperature dependence of number density C_B_ and mean diameter D_B_ of the helium bubble in the Arrhenius plot.

The helium state in the helium bubble can be divided into two limit cases, namely (1) ideal helium or equilibrium bubble, and (2) bubble with constant helium density or overpressure bubble [29]. This difference in helium states has important implications for predicting the behavior of helium bubbles [34]. For example, the smaller the size of a helium bubble is, the greater the internal pressure, and the higher the concentration of He atoms in the surrounding matrix. This concentration gradient will cause the helium atoms to diffuse toward the larger helium bubbles and cause the smaller bubbles to decompose, while the larger bubbles will grow larger [28]. Due to the high permeability of silicon crystals [16], the dissociation and migration of helium atoms are more intense. Even at a lower temperature, the injected helium atoms are also migrating violently. Therefore, different mechanisms may play a role together in the growth process of helium bubbles, resulting in unclear research on the growth mechanism of helium bubbles in monocrystalline silicon. The concrete mechanism of helium bubble nucleation and the diffusion of helium atoms or helium-atom clusters in monocrystalline silicon will be explained by the following analysis. In Figure 7, we plot the relationship between the number density of helium bubbles/the mean diameter of helium bubbles and the reciprocal of the implanted ion temperature. When this relationship is fitted to the Arrhenius behavior, the apparent activation energies of the number density C_B_ and the mean diameter D_B_ of the helium bubble can be calculated [33]. To make our conclusions more concise, we have just selected four key temperature points (115 °C, 145 °C, 175 °C, 220 °C). The growth of helium bubbles at different ion implantation temperatures can be divided into three stages:

115 °C~145 °C:

Apparent activation energy: D_B1_ = 0.02 eV, C_B1_ = 0.17 eV; (1)

145 °C~175 °C:

Apparent activation energy: D_B2_ = 0.27 eV, C_B2_ = 0.4 eV; (2)

175 °C~220 °C:

Apparent activation energy: D_B3_ = 0.28 eV, C_B3_ = 0.2 eV; (3)

The existence of the three states indicates that the mechanism of helium bubble formation varies in the temperature range considered. The calculated apparent activation energy (1) is much less than the dissociation energy released by helium atoms from helium bubbles (1.8 eV in monocrystalline silicon [21,35]), indicating that the formation of helium bubbles is hardly controlled by the thermal decomposition of helium atoms from helium bubbles below 220 °C. This conclusion is consistent with reports in the literature: implantation helium desorption from bubbles occurs for temperatures higher than 700 °C [19] or about 970 K [22]. (2) E^act^ D_B1_ ≤ E^act^ C_B1_ indicates that the helium state in the helium bubble in the 115 °C~145 °C stage is a constant density or overpressure state [32]. E^act^ D_B2_ is slightly smaller than E^act^ C_B2_, indicating that the helium state in the helium bubble is in the transition state of the overpressure helium bubble and the equilibrium helium bubble. E^act^ D_B3_ ≈ E^act^ C_B3_, indicating that the helium state inside the helium bubble may be close to ideal helium and equilibrium bubbles [32]. This change in the helium state indicates that the density of helium atoms inside the helium bubble decreases with increasing temperature. (3) The apparent activation energy E^act^ D_B1_ ≈ 0 indicates that the growth of helium bubbles in the 115 °C~145 °C stage is close to a nonthermal mechanism, which is probably a self-trapping/self-induction mechanism. (4) The apparent activation energies E^act^ D_B2,3_ and E^act^ C_B2,3_ are close to the vacancy migration energy at low temperatures (the vacancy migration energy is approximately 0.37 eV in monocrystalline silicon [36]), so the diffusion mechanism of helium may be a self-interstitial/He displacement mechanism at the 145 °C~220 °C stage. This conclusion can also be discriminated by the following relationship: E^act^ C_B_ = −3 E^M^_V_/7 (E^M^_V_ is the activation energy of vacancy migration, approximately 0.37 eV in monocrystalline silicon [36]) [37]; thus, the apparent activation energy E^act^ C_B_ = 0.16 eV is calculated, and (5) it is close to the apparent activation energy E^act^ C_B3_ = 0.2 eV obtained in our experiments, indicating that the mechanism of helium diffusion in the stage from 175 °C~220 °C should be a self-interstitial/He replacement mechanism. (6), but it is smaller than E^act^ C_B2_ = 0.4 eV, indicating that the growth of helium bubbles in the 145 °C~175 °C stage is also involved in the self-trapping/self-induction mechanism, it can be verified by the following report: helium desorption from small vacancy clusters only for temperature as low as 130 °C [22]. This stage should be a transition state of the self-trapping/self-induction mechanism and the self-interstitial/He replacement mechanism. 

In addition, the physical significance of the activation energy obtained from the experiment can be given by the following equation [38] at the 145 °C~220 °C stage:

lnR_b_ = constant − E^m^_He/_(4 kT) (4)

lnR_b_ = constant − E^m^_He/_(6 kT) (5)

where R_b_ is the radius of the helium bubble, E^m^_He_ is the migration energy of He, and K is Ludwig Boltzmann’s constant. Two different equations may be due to the influence of the state of helium in the bubble [38].

Based on the activation energy of E^act^D_B2,3_ ≈ 0.27 eV obtained for R_b_ in the experiment, E^m^_He_ can be estimated as E^m^_He_ = 0.27 eV × 4 = 1.08 eV and E^m^_He_ = 0.27 eV × 6 = 1.62 eV by Equations (3) and (4), respectively. This result is roughly consistent with E^m^_He_ = 1.34 eV reported by Van et al. in the literature [35]. This similarity indicates that the mechanism of helium diffusion is controlled by the self-interstitial/He replacement mechanism at 145 °C~220 °C.

Combined with the above data analysis, we speculate that the growth mechanism of helium bubbles in this experiment should be as follows: (1) In the temperature range of 115 °C~220 °C, ion implantation brings supersaturated helium atoms and vacancies to the silicon lattice. When the temperature of ion implantation is lower than 175 °C, the number of supersaturated helium atoms is much larger than the number of vacancies, which results in a shortage of vacancies. The supersaturated helium atoms are partly deposited in the tetrahedral interstitial sites of the silicon lattice to form the Si-He complex, that is, interstitial defects-lamellar or rod-like defects. The other part of the helium atoms combine with vacancies to form bubble nuclei, and then the bubble nucleus further captures surrounding helium atoms and vacancies and grows into a helium bubble that can be identified under an electron microscope. (2) With increasing ion implantation temperature, at 145 °C~175 °C, the number of vacancies in the silicon lattice increases, the number of interstitial defects decreases, the number of He-vacancy complexes increases, and the number of bubble nuclei increases. This conclusion is consistent with reports in the literature: irradiation at high temperatures allows for the disappearance of vacancies and self-interstitial atoms and the survival of stable helium-vacancy complexes, which grow up into observable bubbles [22]. The disappearance of the defect may also be due to collisions during helium ion implantation (the L. Pizzagalli groups’ observations suggest that the underlying mechanism is direct helium detrapping through ballistic collisions, leading to the ejection of the helium atoms from the bubble [21]). The smaller the number of supersaturated helium atoms available in the silicon lattice for the growth of a single bubble nucleus, the smaller the bubble diameter. The mechanism of helium bubble growth and helium diffusion in this stage is first a self-trapping/self-inducing mechanism, and with increasing temperature, it becomes a transitional state of the self-trapping/self-inducing mechanism and the self-interstitial/He displacement mechanism. In this stage, the temperature is very low, the diffusion of helium atoms is short-range diffusion, the diffusion of He_m_-V_n_ is more difficult, and nucleation is dominant. Therefore, with increasing ion implantation temperature, the number density of helium bubbles increases, and the diameter of helium bubbles decreases. (3) When the ion implantation temperature is near 175 °C, almost no interstitial defects are observed in Figure 1c, which indicates that the number of vacancies increases to approximately the same as the number of supersaturated helium atoms. Moreover, the combination of the helium atom and vacancy is more favorable to lower the system energy than the combination of the helium atom and silicon lattice. In the temperature range from 175 °C to 220 °C, the mechanism of helium bubble growth and helium diffusion is a self-interstitial/He replacement mechanism. With the increase in temperature, some helium atoms in the bubble nucleus are emitted by self-interstitial recombination, the number of nuclei decreases, the diameter of the observed helium bubble increases, and the density of the number of helium bubbles decreases. 

## 5. Conclusions

In this paper, through the process of helium ion implantation, nanoporous silicon with a 1~5 nm pore size was successfully prepared, control of the pore size change per unit nanometer was realized, and the growth mechanism and regulation mechanism of helium bubble pores in monocrystalline silicon was revealed. A transition state mechanism for helium bubble growth was found.

(1) First, 100 keV helium ions (dose 5 × 10^16^ ions/cm^2^) were implanted into monocrystalline silicon at 115 °C~220 °C. It is found that the growth of helium bubbles is different from the positive correlation with the increase in ion implantation temperature in other reports [19,20], but there are three distinct stages: (i). the 115 °C~145 °C stage, the helium bubble nucleation growth and helium diffusion mechanisms are self-trapping/self-induction mechanisms. With increasing ion implantation temperature, the diameter of the helium bubbles is unchanged, and the number density of the helium bubbles increases. (ii). at the 145 °C~175 °C stage, the helium bubble nucleation growth and helium diffusion mechanism are a transition state of self-trapping/self-induction mechanism and self-interstitial/He replacement mechanism. With increasing ion implantation temperature, the diameter of the helium bubble decreases, and the helium bubble number density increases. (iii). from 175 °C to 220 °C, the helium bubble nucleation growth and helium diffusion mechanism is a self-interstitial/He replacement mechanism. With increasing ion implantation temperature, the diameter of the helium bubbles increases, and the number density of the helium bubbles decreases. At a temperature of approximately 175 °C, the smallest helium bubble diameter is approximately 2.3 nm, and the largest helium bubble number density is 4.2 × 10^23^ m^−3^. In this process, the porous structure is not expected to be obtained when the injection temperature is lower than 115 °C.

(2) At 180 °C, the diameter of the helium bubble increased by 57%, and the number density of the helium bubble increased by 33% with increasing helium ion implantation dose from 2.5 × 10^16^ ions/cm^2^ to 7.5 × 10^16^ ions/cm^2^. There is a critical concentration for the formation of helium bubbles in monocrystalline silicon, which is between 1 × 10^16^ ions/cm^2^ and 2.5 × 10^16^ ions/cm^2^, rather than the critical dose of 1 × 10^16^ ions/cm^2^ reported in the literature [16] for helium bubble formation.

## Figures and Tables

**Table 1:** Helium bubble growth under different helium ion implantation.

Sample Number	Ion Implantation Temperature (°C)	Implanted Ion Energy (KeV)	Implanted Ion Dose (Ions × cm^−2^)	Defect Layer Width (±20 nm)	Average Helium Bubble Diameter (±0.5 nm)	Helium Bubble Number Density (±0.5 × 10^23^ m^−3^)	Other Defects
0							
1	115	100	5 × 10^16^	450	4	1.4	A large number of sheet-like defects
2	145	100	5 × 10^16^	440	3.8	2	A large number of sheet-like defects
3	160	100	5 × 10^16^	450	2.8	3.6	
4	175	100	5 × 10^16^	450	2.3	4.2	
5	190	100	5 × 10^16^	450	3.8	3.2	
6	220	100	5 × 10^16^	480	4.5	2.6	
7	180	100	1 × 10^16^				
8	180	100	2.5 × 10^16^	450	3	2.2	
9	180	100	5 × 10^16^	450	3.7	2.2	
10	180	100	7.5 × 10^16^	450	4.7	2.6	

## Data Availability

Not applicable.
